# Supplementary figures and images for: Molecular and Pathological Characterization of Classical Swine Fever Virus Genotype 2 Strains Responsible for the 2013–2018 Outbreak in Colombia
Source: Viruses. 2023 Nov 24;15(12):2308. doi: 10.3390/v15122308 (PMC10747092; doi:10.3390/v15122308)

Region

- Africa
- Americas
- Asia
- Europe

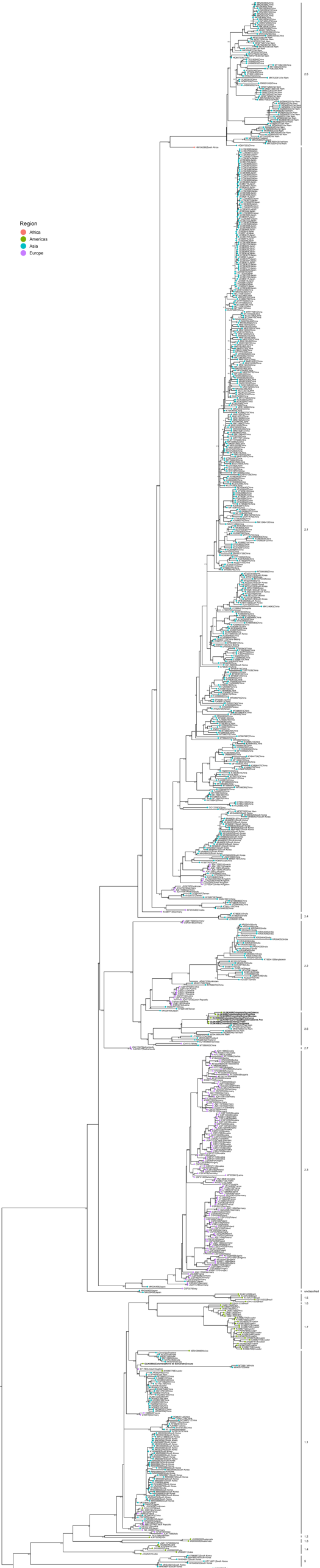

Supplement: Supplementary file 1 [file viruses-15-02308-s001.zip › viruses-2617177-supplementary.pdf]
